# The CoxD Protein, a Novel AAA+ ATPase Involved in Metal Cluster Assembly: Hydrolysis of Nucleotide-Triphosphates and Oligomerization

**DOI:** 10.1371/journal.pone.0047424

**Published:** 2012-10-15

**Authors:** Tobias Maisel, Stephanie Joseph, Thorsten Mielke, Jörg Bürger, Stephan Schwarzinger, Ortwin Meyer

**Affiliations:** 1 Chair of Microbiology, University of Bayreuth, Bayreuth, Germany; 2 Max-Planck-Institute for Molecular Genetics, Berlin, Germany; 3 Charité-Universitätsmedizin Berlin, Institute of Medical Physics and Biophysics, Berlin, Germany; 4 Chair of Biopolymers, University of Bayreuth, Bayreuth, Germany; 5 The Bayreuth Research Center for Bio-Macromolecules, Bayreuth, Germany; University of Cambridge, United Kingdom

## Abstract

CoxD of the α-proteobacterium *Oligotropha carboxidovorans* is a membrane protein which is involved in the posttranslational biosynthesis of the [CuSMoO_2_] cluster in the active site of the enzyme CO dehydrogenase. The bacteria synthesize CoxD only in the presence of CO. Recombinant CoxD produced in *E. coli* K38 pGP1-2/pETMW2 appeared in inclusion bodies from where it was solubilized by urea and refolded by stepwise dilution. Circular dichroism spectroscopy revealed the presence of secondary structural elements in refolded CoxD. CoxD is a P-loop ATPase of the AAA-protein family. Refolded CoxD catalyzed the hydrolysis of MgATP yielding MgADP and inorganic phosphate at a 1∶1∶1 molar ratio. The reaction was inhibited by the slow hydrolysable MgATP-γ-S. GTPase activity of CoxD did not exceed 2% of the ATPase activity. Employing different methods (non linear regression, Hanes and Woolf, Lineweaver-Burk), preparations of CoxD revealed a mean K_M_ value of 0.69±0.14 mM ATP and an apparent V_max_ value of 19.3±2.3 nmol ATP hydrolyzed min^−1^ mg^−1^. Sucrose density gradient centrifugation and gel filtration showed that refolded CoxD can exist in various multimeric states (2-mer, 4-mer or 6-mer), preferentially as hexamer or dimer. Within weeks the hexamer dissociates into the dimer, a process which can be reversed by MgATP or MgATP-γ-S within hours. Only the hexamers and the dimers exhibited MgATPase activity. Transmission electron microscopy of negatively stained CoxD preparations revealed distinct particles within a size range of 10–16 nm, which further corroborates the oligomeric organization. The 3D structure of CoxD was modeled with the 3D structure of BchI from *Rhodobacter capsulatus* as template. It has the key elements of an AAA+ domain in the same arrangement and at same positions as in BchI and displays the characteristic inserts of the PS-II-insert clade. Possible functions of CoxD in [CuSMoO_2_] cluster assembly are discussed.

## Introduction

The product of the *coxD* gene in the Gram-negative, chemolithoautotrophic α-proteobacterium *Oligotropha carboxidovorans* OM5 [1] functions in the posttranslational introduction of sulfur and copper into a [MoO_3_] site during the biosynthesis of the [CuSMoO_2_] active site cluster of the enzyme CO dehydrogenase [2]. CO dehydrogenase is a structurally characterized iron-sulfur flavoprotein which uniquely combines Mo- and Cu-ion in its active site [3], [4] and catalyzes the oxidation of CO (CO + H_2_O → CO_2_+2 e^−^ +2 H^+^ [5]). The electrons thereby produced fuel the translocation of protons by a respiratory chain to generate a proton motive force across the cytoplasmic membrane. Thereby the bacteria are supplied with the energy required for the fixation of CO_2_ in the CBB-cycle [6]. Disruption of *coxD* in the *coxD* mutant (OM5 D::km) abolished the ability to utilize CO, indicating a role of CoxD in the CO metabolism of the bacterium [2]. The apo-CO dehydrogenase synthesized in bacteria grown chemolithoautotrophically with H_2_, O_2_ and CO_2_, in the presence of CO as an inducer of enzyme biosynthesis, contained a [MoO_3_] site in place of the complete [CuSMoO_2_] cluster required for catalysis [2]. This indicated a function of the *coxD* gene product in cluster biosynthesis.

The *coxD* gene is one of 12 genes included in the 14.5-kb *cox*-gene cluster on the low-copy number, circular 133-kb DNA megaplasmid pHCG3 of *O. carboxidovorans* [7]. The occurrence of *cox* genes in bacteria of quite distinct phylogenetic positions [8] suggests that they are conserved and presumably subject to a horizontal transfer. C*ox* genes code for the CO dehydrogenase polypeptides (CoxM, CoxS, and CoxL) as well as for CoxD, CoxE and CoxF which are engaged in the biosynthesis of the [CuSMoO_2_] cluster [9], [2]. The prominent clustering of *cox*-genes is also apparent in other bacteria (e.g. *Mycobacterium tuberculosis*, *Bradyrhizobium japonicum* or *Hydrogenophaga pseudoflava*), and *coxDEF* are always grouped together immediately downstream of *coxMSL*, although their individual orders might differ [8]. The *cox*-cluster along with the *cbb*-cluster on pHCG3 are transcribed in a CO-dependent manner [8], [2].

**Figure 1:**
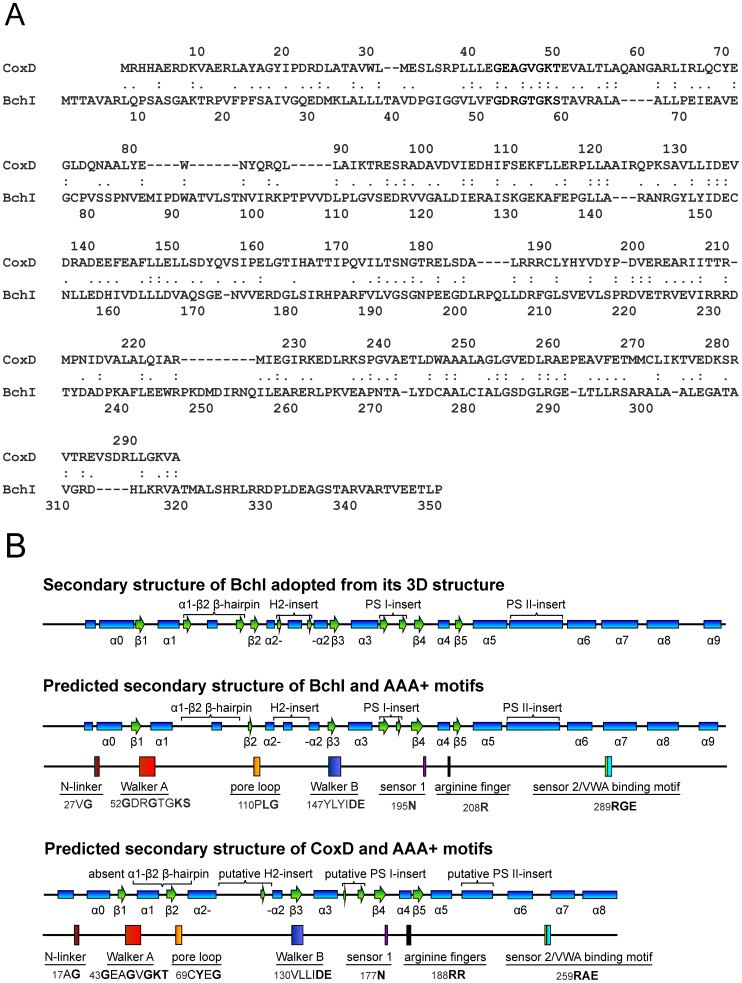
Sequence similarity between CoxD of *Oligotropha carboxidovorans* and BchI of the *Rhodobacter capsulatus* Mg-chelatase complex (*top*) along with secondary structural elements and AAA+ motifs in CoxD and BchI (*bottom*). Panel (A) compares the sequences of CoxD (295 aa) of *O. carboxidovorans* and BchI (350 aa) of *R. capsulatus* employing the UVa FASTA Server (fasta.bioch.virginia.edu/fasta_www2/fasta_list2.shtml). Colons and dots indicate identity or similarity, respectively. The secondary structures shown in panel (B) have been adopted from the crystal structure of BchI [31] and the amino acid sequence of CoxD [2], [7], [36], [47]. The N-terminus of CoxD contains the N-linker showing the characteristic motif consisting of a hydrophobic amino acid (A^17^) and glycine (G^18^). The P-loop of the Walker A motif lies between strand β1 and helix α2 and contains the consensus G^43^XXGXG^48^KT^50^, whereas V^130^LLIDE^135^ on β3 of CoxD is the Walker B motif (hhhhDE, h stands for hydrophobic aa) which interacts with ATP. The loop between β2 and α2 is the suggested pore loop of CoxD. The conserved aromatic-hydrophobic-glycine pore loop motif of AAA unfoldases/translocases that resides between β2 and α2 of the AAA+ core [49] is modified in BchI to PLG. The second region of homology (SRH) is considered to coordinate nucleotide hydrolysis and conformational changes between subunits of the AAA ATPase oligomer [48]. SRH of CoxD (aa 175–189) consists of part of β4 and all of α4 and contains sensor 1 (N^177^) and arginine fingers (R^188^R^189^) at the end of α4. Sensor 2 (R^259^) of CoxD resides near the N-terminal end of α7 and is as in BchI part of the VWA-binding motif (R^259^AE^261^). The numbers preceding the motifs in panel (B) refer to the position of the first amino acid. Blue rectangles refer to α-helices, and green arrows indicate β-strands.

CoxD is a predicted member of the class of AAA+ ATPase chaperones (ATPases associated with a variety of cellular activities [2], [10], [11]). CoxD has been positioned in a cluster of MoxR-like sequences of the PACTT group (proteases, chelatases, transcription activators, and transport proteins; [12]) and in the PS-II insert clade which among others contains dynein, midasin, and chelatases, e.g. Mg^2+^-chelatases [13]. Mg^2+^-chelatases assemble in form of protein complexes which catalyze the first committed step of the chlorophyll biosynthetic pathway of plants and bacteria, the ATP-dependent insertion of Mg^2+^ into protoporphyrin IX (for a review see [14]). Mg^2+^-chelatase seems to be present in all photosynthetic organisms, including plants and bacteria. The enzyme complex consists of different protein subunits, referred to as BchI, BchD and BchH, in organisms that synthesize bacteriochlorophylls and ChlI, ChlD and ChlH in organisms that synthesize chlorophylls [15], [14]. The Mg^2+^-chelatase subunits from different sources are similar in sequences and sizes [15], [14], and CoxD and CoxE resemble some of them; the BchI/ChlI homologoues being ∼40 kDa (CoxD ∼ 33.3 kDa), the BchD/ChlD homologues being ∼65 kDa (CoxE ∼ 44 kDa) and the BchH/ChlH homologous being ∼140 kDa (unrelated to the ∼ 29 kDa CoxF in sequence and size). Mg^2+^-chelatase activity requires ATP, Mg^2+^, protoporphyrin IX and all three subunits of the complex [14]. CoxD is closely related to the hexameric, ring-shaped BchI component of the Mg^2+^-chelatase of *Rhodobacter capsulatus*. The sequence of CoxD gives blast scores of 22.0%, 20.7%, or 19.5% identity and 49.2%, 46.6%, or 47.1% similarity with BchI of *Rhodobacter capsulatus*, *Rhodobacter sphaeroides*, or *Synechocystis* PCC6803, respectively (Fig. 1A). CoxD comprises all of the characteristic key elements of BchI (Fig. 1B). Particularly, the Walker A and Walker B motifs on the CoxD amino acid sequence suggest the ability to bind and hydrolyze MgATP. Higher complex formation of CoxD and CoxE can be easily imagined through interaction of the VWA/MIDAS domain on CoxE with the VWA-binding motif on CoxD ([2], Fig. 1). The anticipated function of CoxD is the MgATP-dependent partial unfolding of apo-CO dehydrogenase to assist in the stepwise introduction of S and Cu in the enzymés [MoO_3_] center.

In this report the non-tagged CoxD protein was heterologously expressed in *E. coli* as inclusion bodies. The latter were purified and solubilized in urea. CoxD was refolded by dilution. Refolded CoxD has been studied with respect to AAA+ ATPase properties, particularly nucleotide-triphosphate hydrolysis, oligomerization, and its possible three-dimensional structure in relation to BchI of Mg^2+^-chelatase.

## Materials and Methods

### Cultivation of *O. Carboxidovorans*

Wild-type and mutant strains were grown in 70-liter fermentors (model Biostat, Braun Melsungen, Germany) in a mineral medium [16]. For chemolithoautotrophic growth the fermentors were supplied with (v/v) 45% CO, 5% CO_2_, and 50% air, or 30% CO, 30% H_2_, 5% CO_2_, and 35% air, or 40% H_2_, 10% CO_2_, and 50% air, respectively. For heterotrophic growth 0.2% (w/v) Na-pyruvate and 0.3% (w/v) nutrient broth were added to the mineral medium. After the cell-harvest in the late exponential phase, the bacteria were stored at −80°C until use.

**Figure 2:**
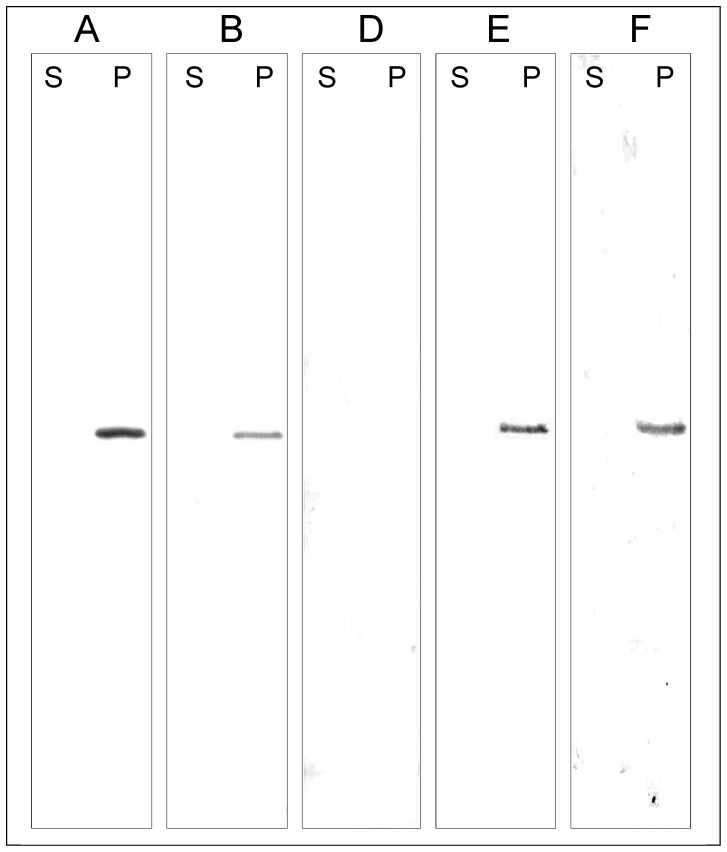
Detection of CoxD in subcellular fractions of *O. carboxidovorans* cultivated under different conditions. *O. carboxidovorans* OM5 (lanes A and B) and the insertional mutants *coxD* (lane D), *coxE* (lane E), and *coxF* (lane F) were grown under different metabolic conditions: *lane A*, chemolithoautotrophically with CO (45 CO, 5 CO_2_, 50 air; all values in % v/v); *all other lanes*, chemolithoautotrophically with H_2_ in the presence of CO (30 H_2_, 5 CO_2_, 30 CO, 35 air) to induce the transcription of *cox*-genes. In order to separate cytoplasmic fractions (S, supernatant) and membrane fractions (P, pellet), crude extracts prepared from the bacteria were subjected to ultracentrifugation (100,000× *g*, 2 h, and 4°C). The distribution of CoxD in the supernatant or the pellet was analyzed by applying 50 µg of protein from S or P to SDS-PAGE. Prior to PAGE all samples were boiled in 1% SDS as described in the methods section. CoxD was identified by Western blotting employing IgG antibodies raised against recombinant CoxD. The grey bands apparent from lanes A, B, E, and F originate from the CoxD protein.

### Production of Heterologous CoxD in *E. coli*

The construct *E. coli* K38 pGP1-2/pETMW2, which carries the *coxD* gene on the plasmid pETMW2 [2], was cultivated aerobically at 30°C in a 30-l fermentor supplied with 20 l of LB medium [17] supplemented with ampicillin (200 mg l^−1^) and kanamycin (100 mg l^−1^). The expression of CoxD was induced at an optical density (OD_600_) of 0.7 with 1 mM isopropyl-β-D-thiogalactopyranoside (IPTG) and a subsequent heat shock (1.5 h at 42°C). Due to the high-level expression, CoxD appeared entirely in inclusion bodies. Crude extracts were centrifuged to sediment the inclusion bodies (9,000×*g*, 4°C, 30 min). Contaminating material was removed from the inclusion bodies employing two washing steps. For that purpose they were homogenized in 20 mM Tris/HCl (pH 8.0) supplemented with 1% Triton X-100 and 150 mM NaCl, and centrifuged as above. Finally, the detergent was removed by two washes in Tris/HCl.

**Figure 3:**
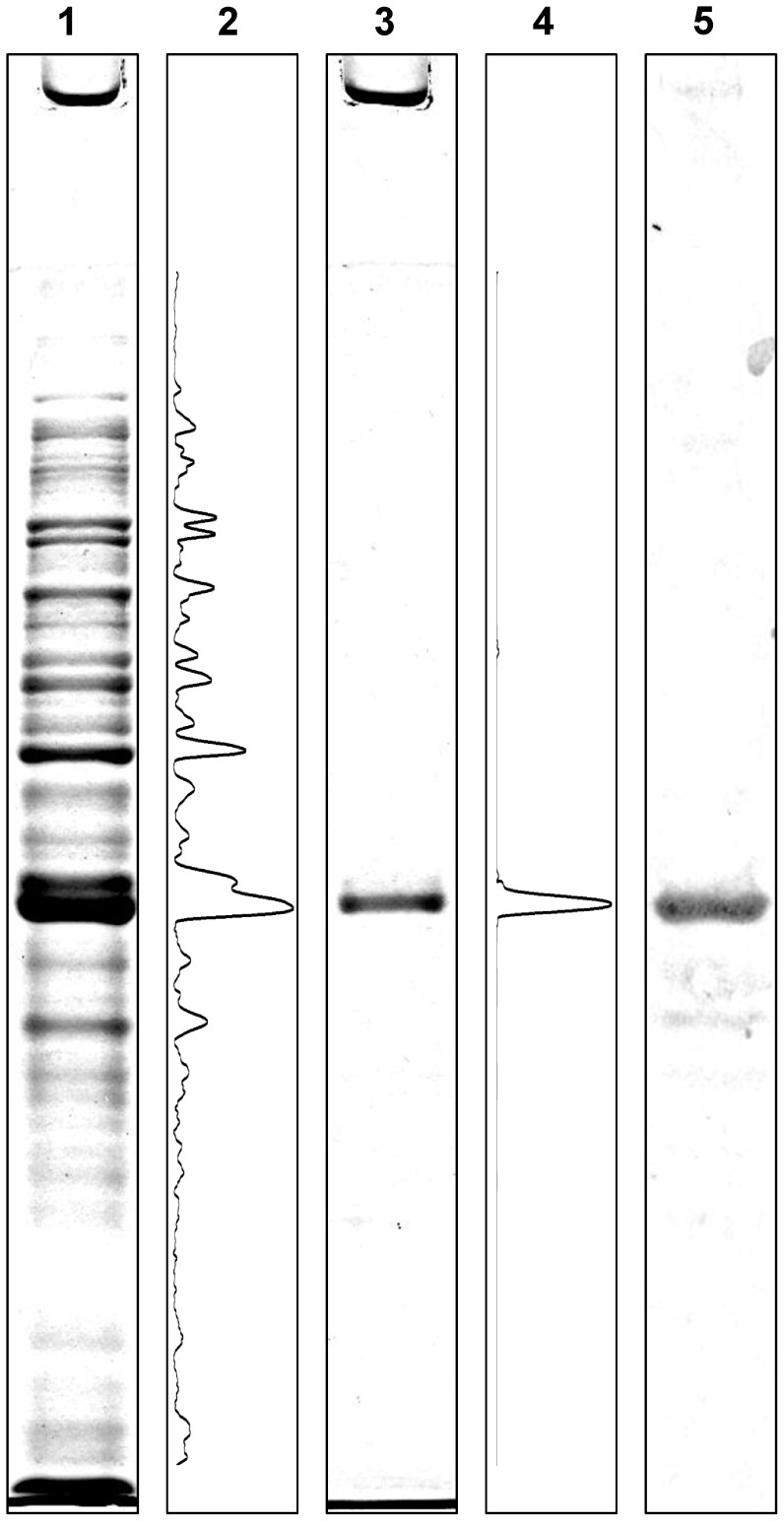
Analysis of recombinant CoxD by SDS-PAGE. The formation of CoxD in *E. coli* K38 pGP1-2/pETMW2 was induced with 1 mM IPTG followed by a heat shock (42°C). Intact bacterial cells or washed inclusion bodies suspended in Tris/HCl supplied with SDS and β-mercaptoethanol were boiled for 4 min as detailed in Materials and Methods. Lanes 1 and 3 received 30 µg of bacterial cell protein or 5 µg of inclusion body protein, respectively. Protein was stained with Coomassie Brilliant Blue. The program ImageJ (lanes 2 and 4) was employed for densitometry [33]. Lane 5 represents the same experiment as in lane 1, except that 5 µg of bacterial cell protein was applied and analysis was by Western-blotting employing IgG antibodies directed against CoxD.

### Solubilization and Refolding of CoxD

In order to solubilize CoxD, washed inclusion bodies were suspended in a mixture composed of 8 M urea, 10 mM mercaptoethanol, 10 mM dithiothreitol, 1 mM reduced glutathione, 1 mM Na_2_EDTA, 1 mM glycine, and 100 mM Tris and stirred magnetically at 4°C for 1 h. None solubilized material was removed by centrifugation (25,000× *g*, 4°C, 60 min) followed by ultra-filtration employing polyvinylidene fluoride membranes (0.22 µm). Solubilized CoxD was refolded by ∼50-fold dilution employing pulsed renaturation. For that purpose, the following standard protocol or variations thereof was employed. CoxD solution (60 ml) was added drop wise (∼ 50 µl) to 3,000 ml of ice cold, filtered, magnetically stirred 20 mM Tris. Immediately after the dilution step the pH was adjusted to 9.0 with 1 N HCl and kept for 24 h at 4°C. Then, the pH was gradually decreased to 8.0 in increments of 0.2 per 24 h. The resulting solution composed of 2 mM Tris, 160 mM urea, 200 µM β-mercaptoethanol, 200 µM DTT, 20 µM reduced glutathione, 20 µM EDTA, and 20 µM glycine is being referred to as refolding buffer. Aggregation of CoxD in the course of refolding was checked regularly through the inspection of its UV/VIS-absorption spectrum for the appearance of turbidity.

**Figure 4:**
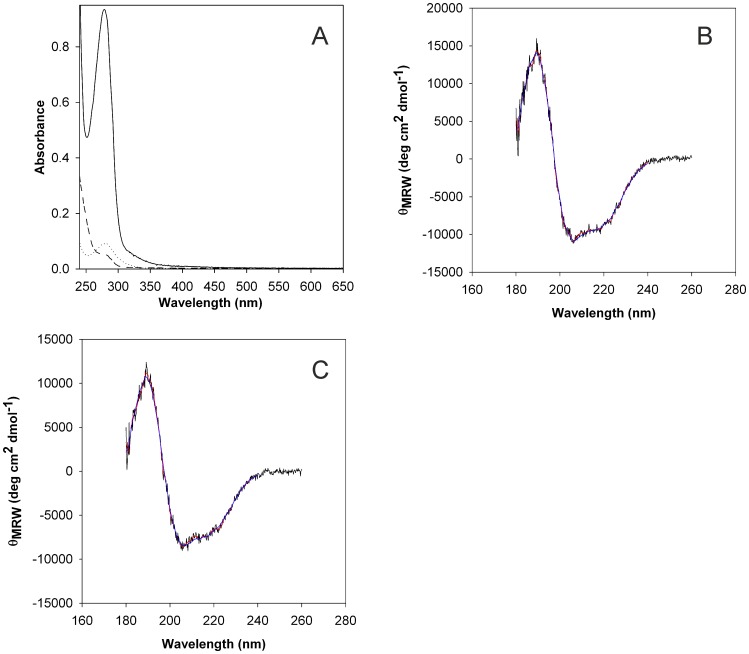
Absorption spectra (A) and circular dichroism (CD) spectra of refolded CoxD kept in the absence (B) or presence of ATP-γ-S (C). Solubilized CoxD (5.5 mg ml^−1^) was refolded by rapid dilution (50-fold) in ice cold aqueous Tris (20 mM) under magnetic stirring. The solution was immediately adjusted to pH 9.0 (A, dashed line) and subsequently brought to pH 8.0 (A, dotted line) in pH increments of 0.2 per 24 h as specified in Materials and Methods. Then the protein was concentrated to 1.04 mg ml^−1^ by ultra-filtration (A, solid line). The CD spectra of CoxD (0.471 mg ml^−1^; 10 mM KH_2_PO_4_/KOH, pH 8.0) are shown in (B). The CD spectra of CoxD (0.537 mg ml^−1^; 10 mM KH_2_PO_4_/KOH, pH 8.0) in the presence of 0.1 mM MgATP-γ-S are shown in (C). After 2 h of incubation with MgATP-γ-S, excess nucleotides were removed by gel filtration on Sephadex G-25. All data were recorded at 20°C. Raw data are shown in black. The smoothed data used for secondary structure estimation and the back-calculated CD-spectrum based on deconvolution with the CDSSTR algorithm [21] is depicted in red and blue, respectively. For further details refer to Materials and Methods.

### Circular-Dichroism Spectroscopy

CoxD in 10 mM KH_2_PO_4_/KOH (pH 8.0) contained in a quartz cell (d = 0.1 cm; Hellma Analytics, Mühlheim, Germany) was positioned under N_2_ in a circular-dichroism (CD) spectrometer (model J810, Jasco Germany, Groß-Umstadt, Germany) equipped with an Jasco CDF-426S Peltier thermostat, set to 20°C. CD spectra were acquired from 176 nm to 260 nm using the step mode with a step size of 0.2 nm, a band width of 1 nm, and an integration time of 2 s. Solvent spectra, obtained under same conditions, were subtracted and the data were smoothed using a Savitzky Golay filter [18], with a window size of 17 data points. Spectra were converted to residual mean molar ellipticity (molecular mass of 33,269 Da, 295 amino acids) and the secondary structure content was determined with the online server Dichroweb [19] employing the SP175 [20] reference data set and the CDSSTR algorithm [21].

**Figure 5:**
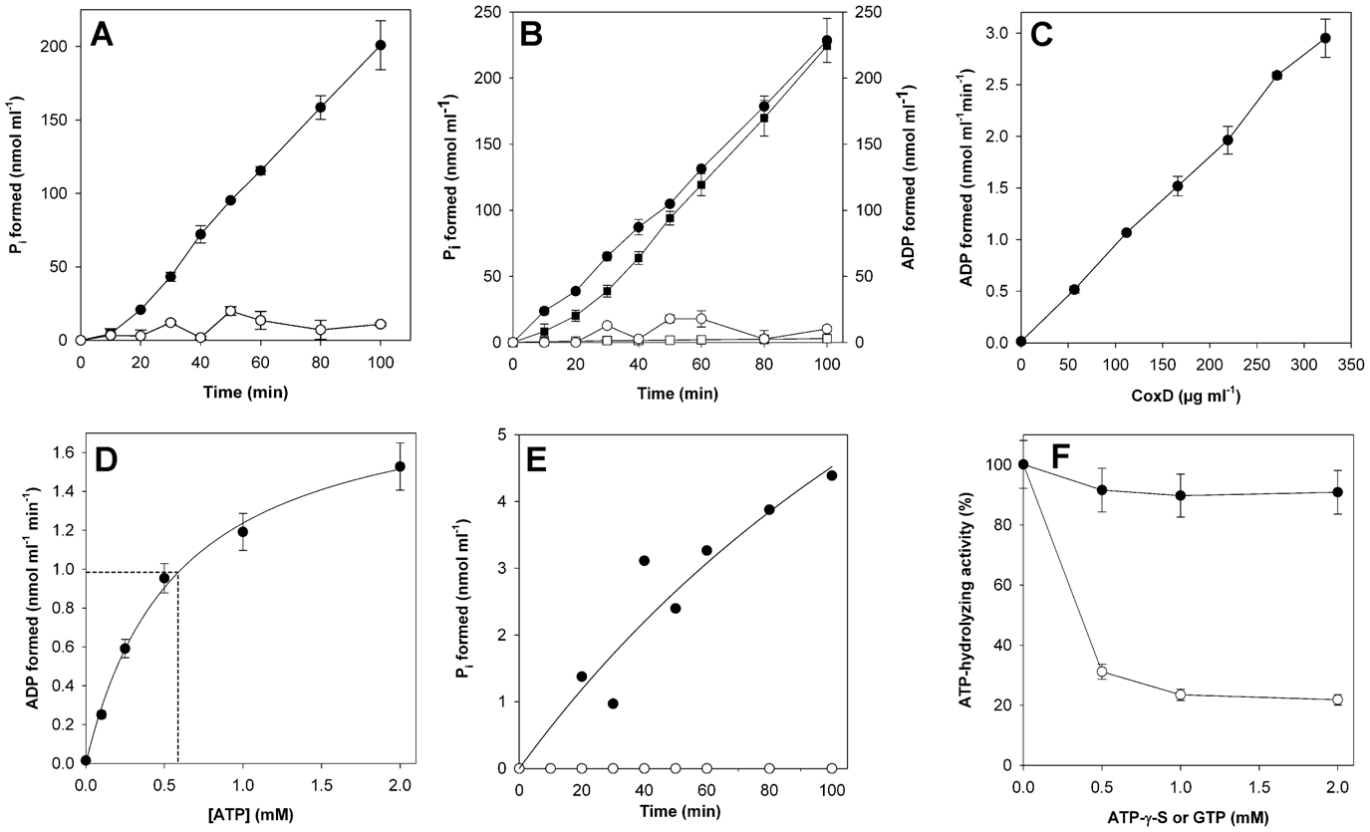
Hydrolysis of NTPs by refolded CoxD. (A) Release of P_i_ from ATP (1 mM Na_2_ATP in 100 mM Tris/acetate, pH 8.0) catalyzed by CoxD (0.22 mg protein ml^−1^) in the presence (•) or absence (○) of 1 mM Mg^2+^-acetate. (B) Formation of P_i_ (•, ○) and ADP (▪, □) in the presence (•, ▪) or absence (○, □) of CoxD; assays contained 1 mM Mg^2+^-acetate. (C) Correlation of ATPase activity and amount of CoxD (•); assays contained 1 mM Mg^2+^-acetate; incubations were for 60 min. (D) Dependence of the CoxD ATPase activity (0.11 mg protein ml^−1^) on the concentration of ATP in the assay (•). The dotted lines indicate the calculated K_M_ and 0.5 V_max_. (E) Formation of P_i_ from GTP (1 mM Na_2_GTP in 100 mM Tris/acetate, pH 8.0; assays contained 1 mM Mg^2+^-acetate) in the presence (•) or absence (○) of CoxD (0.22 mg protein ml^−1^). (F) Effect of ATP-γ-S (○) or GTP (•) on the hydrolysis of ATP (1 mM) by CoxD. Assays contained 1 mM Mg^2+^-acetate. An activity of 100% corresponds to 9.37 nmol ADP released min^−1^ mg^−1^ (○) or 8.72 nmol ADP released min^−1^ mg^−1^ (•). See Material and Methods for experimental details. All experiments were at 25°C. Experimental errors are indicated by vertical bars.

### Assay of NTP Hydrolyzing Activity

NTPase activity was assayed at 25°C in 100 mM Tris/acetate (pH 8.0) supplemented with 1 mM magnesium acetate and 1 mM disodium adenosine-5′-triphosphate (Na_2_ATP) or 1 mM disodium guanosine-5′-triphosphate (Na_2_GTP). The hydrolysis of MgATP or MgGTP with time was assayed in two ways. The inorganic phosphate released was determined according to published procedures [22]. Alternatively, ATPase activity was followed photometrically (ε_340, NADH_ = 6.22 mM^−1^ cm^−1^) employing a modified MgATP-recycling enzyme linked assay [23]. Assays contained 100 mM Tris/acetate, pH 8.0, 1 mM Na_2_ATP, 1 mM Mg-acetate, 2 mM phosphoenolpyruvate, 0.2 mM nicotinamide adenine dinucleotide (NADH), pyruvate kinase (1.75 U ml^−1^), and lactate dehydrogenase (2.5 U ml^−1^). Reactions were started through the addition of refolded CoxD.

**Figure 6:**
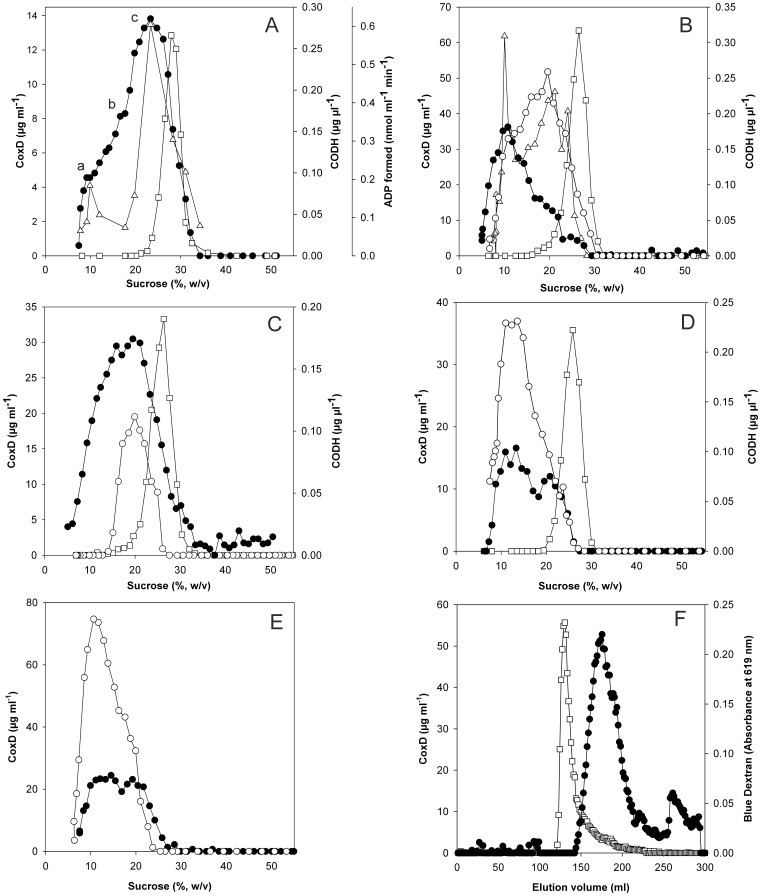
Analysis of oligomerization of refolded CoxD protein by sucrose density gradient centrifugation. CoxD in refolding buffer was subjected to sucrose density gradient centrifugation, and fractions were analyzed as indicated. Coomassie Brilliant Blue was employed for protein estimation. CO dehydrogenase (□) was included as a reference for molecular mass (277,074 Da). For details refer to Materials and Methods. (A) Oligomerization of CoxD and ATPase activity. Sucrose gradients received 1 ml of CoxD (0.5 mg). After centrifugation and fractionation, CoxD protein was determined (•); a, b, and c refer to different oligomers of CoxD, which are discussed in the text. ATPase activity was assayed by following the hydrolysis of MgATP to MgADP (▵) as detailed in the methods section. (B) Stability of CoxD hexamers in diluted and concentrated solution. Sucrose gradients received the following samples: Freshly refolded CoxD (0.01 mg ml^−1^) was concentrated by ultra-filtration (0.9 mg ml^−1^). From this solution, 1.5 mg protein was subjected to sucrose gradient centrifugation (○). Concentrated CoxD was kept for 28 d at 4°C and analyzed again (▵). Freshly refolded CoxD (0.01 mg ml^−1^) was also kept for 28 d at 4°C, concentrated (0.82 mg ml^−1^), and 1.5 mg were subjected to sucrose gradient centrifugation (•). (C) Recentrifugation of hexameric CoxD. Refolded CoxD was concentrated (2.13 mg ml^−1^), and 1.5 mg were applied to sucrose density gradient centrifugation (○). The peak fractions representing the hexamer (19.6% to 22.1% sucrose) of five experiments were pooled. After removal of sucrose by gel filtration employing 100 mM Tris/acetate and concentration (0.21 mg ml^−1^), 0.4 mg CoxD was recentrifuged (•). (D) Impact of ATP on dimeric CoxD. Dimeric CoxD was prepared from the hexamer as described in (B), concentrated (1.5 mg ml^−1^), and 1 mg was subjected to sucrose gradient centrifugation (○). The effect of ATP was examined in samples of 0.5 mg CoxD in 73 mM Tris/acetate (pH 8.0) supplemented with 0.1 mM MgATP. Assays were kept for 2 h at 4°C and then supplied to sucrose density gradients which were amended with 0.1 mM MgATP (•). (E) Impact of slow hydrolysable ATP-γ-S on dimeric CoxD. Conditions and symbols are as in (D) with the exception that the CoxD sample contained 0.1 mM MgATP-γ-S, and the sucrose gradient was devoid of nucleotides. (F) Gel filtration of refolded CoxD (6 mg) on Sephacryl S-300 (•); Blue Dextran (□) served as a void volume marker in a separate run.

**Figure 7:**
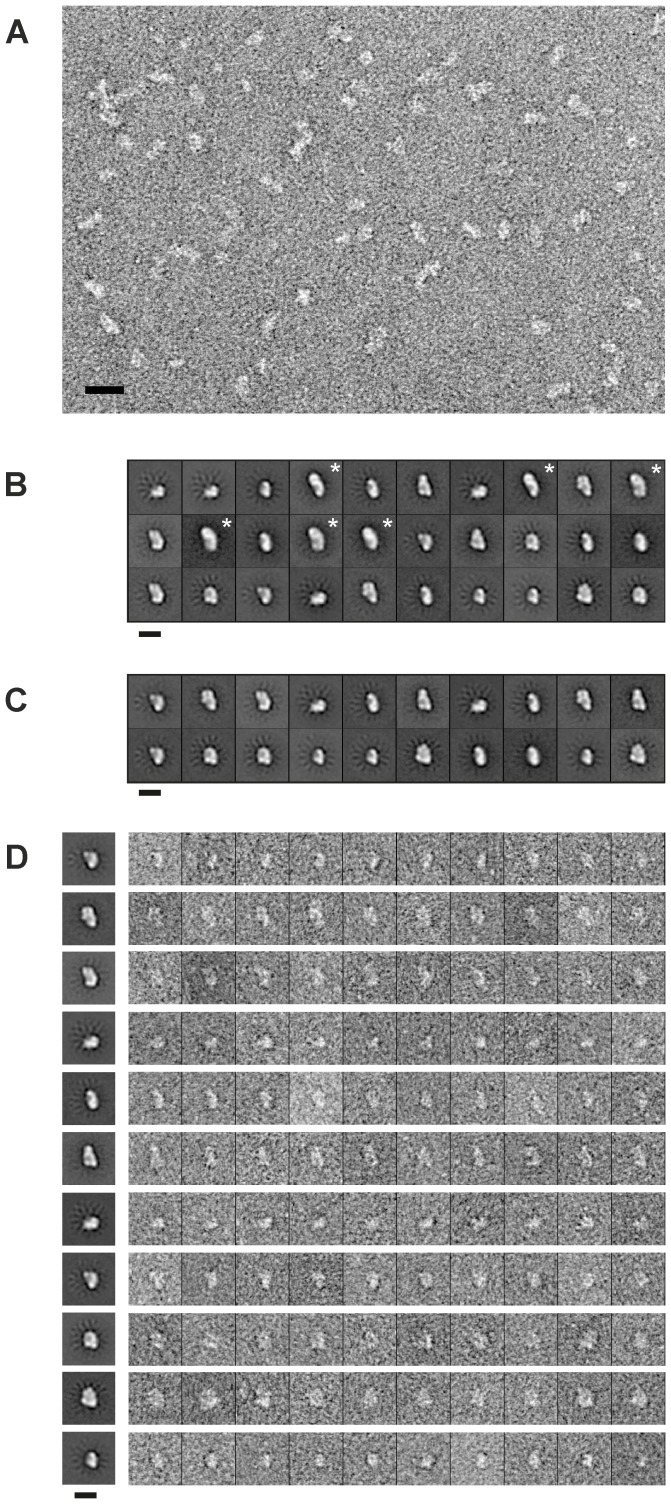
TEM analysis of recombinant CoxD. (A) TEM image of CoxD negatively stained with uranyl acetate. Distinct particles are clearly visible. Most particles exhibit a size of 10–16 nm. Additionally, some smaller fragments and some elongated particles were also present. Scale bar: 25 nm. (B) Initial class averages of CoxD calculated from 19,091 raw particles. Reproducible substructures can be identified. Closer inspection of the classes revealed, that classes showing elongated features (marked with an asterisk) were clearly heterogeneous and were thus rejected from further analysis. (C) Final class averages calculated from 15,876 selected raw particles. Reproducible structure is clearly enhanced, revealing two types of particle views. The larger type of views comprises particles with a length of about 16 nm with a narrow top and wider end, respectively. The second type extents to only 10–11 nm. However, at this level of 2D analysis, it is impossible to assign the views to complexes with different sizes, e.g. to dimers, tetramers and hexamers, respectively, and/or different orientation of the complexes on the grid. (D) Representative class averages (left) and representation of randomly selected members from these classes (right). Scale bar (B–D): 15 nm.

### Sucrose Density Gradient Centrifugation and Gel Filtration

Linear sucrose gradients [84 ml, 5 to 55% (w/v) in 100 mM Tris/acetate, pH 8.0] were formed in polycarbonate tubes. Refolded protein solutions were concentrated by ultra-filtration at 4°C employing membranes with a nominal molecular weight limit of 5,000 (Amicon 8050 and 8400 equipped with Biomax PB polyethersulfone or Ultracel YM cellulose membranes Millipore, Schwalbach, Germany), layered on top of gradients and centrifuged for 20 h at 130,000× *g* and 4°C (Uvikon, Kontron, Eching, Germany). As a reference for molecular mass, a sucrose gradient in a separate tube received 2.5****mg of CO dehydrogenase of *O. carboxidovorans* (277,074 Da) and was included in the same run. After fractionation the contents of protein [34] and sucrose (refractometer, Zeiss, Oberkochen, Germany) were determined. Molecular weights were calculated according to Schachmann [24]. Where indicated, sucrose was removed from samples by gel filtration on Sephadex G-25 (PD-10 pre-packed columns, GE Healthcare, Little Chalfont, UK). Gel filtration on Sephacryl S-300 (separation range 10 kDa –1,500 kDa; Sigma, Taufkirchen, Germany) was in chromatography columns (600 mm×52 mm, model XK26/60, GE Healthcare, Pharmacia, Freiburg, Germany) employing 100 mM Tris/acetate (pH 8.0) as the eluent. The void volume was determined with 6 mg Blue Dextran 2000 (exclusion limit 2000 kDa, GE Healthcare, Pharmacia, Freiburg, Germany) in a separate run. *O. carboxidovorans* CO dehydrogenase (277,074 Da), yeast alcohol dehydrogenase (147,000 Da), bovine liver catalase (247,000 Da), and bovine serum albumin (67,000 Da) were used as molecular mass standards.

**Figure 8:**
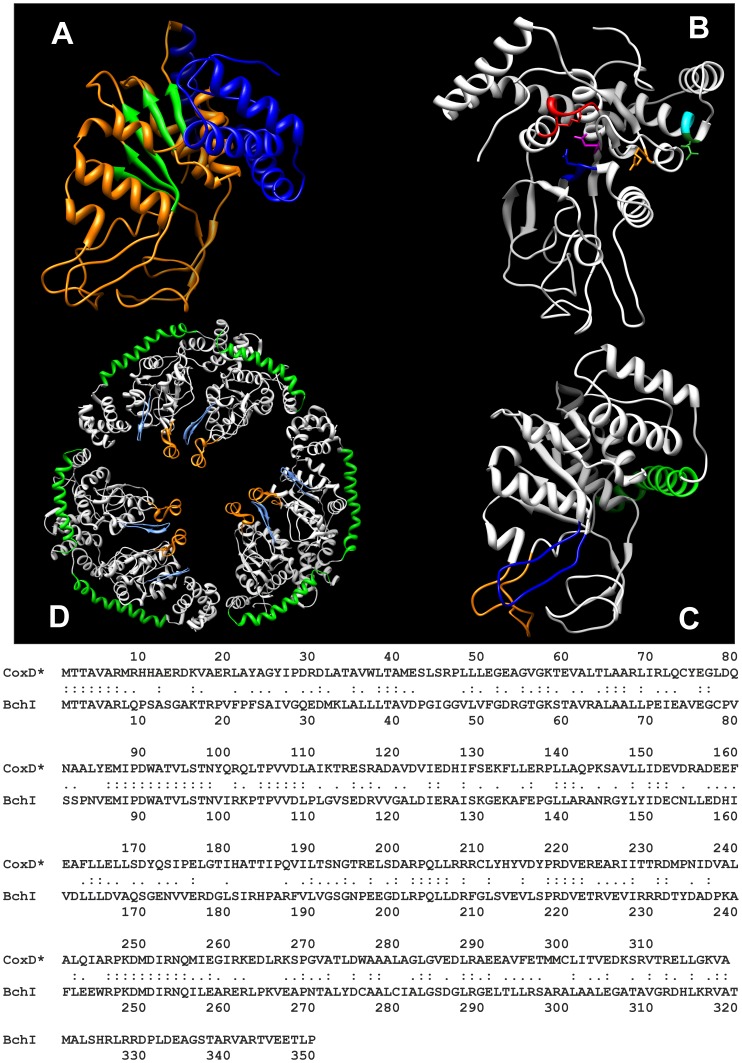
Homology-built model of CoxD (top) in ribbon representation and the sequence alignment (CoxD*) from which the model was build (bottom). The following adjustments were introduced into the amino acid sequence of CoxD to account for differences between CoxD (295 aa) and BchI (350 aa) which interfered with model building by the program ESyPred3D. The N-terminus of CoxD was extended with the sequence M^1^TTAVAR^7^ of BchI (*cf.* Fig. 1). With the exception of the C-terminus ^320^T to ^350^P, all gaps in CoxD relative to BchI were filled up with corresponding BchI sequences (*cf.* Fig. 1): ^39^T^40^A, ^87^MIP^90^D, ^92^ATVLS^97^T, ^104^TPVV^108^D, ^202^RPQ^205^L, ^219^R, ^232^D, ^247^PKDMDIRN^255^Q. The following deletions were made in CoxD to account for gaps in BchI: ^58^QAN^61^G, ^122^AI^124^R, ^156^V, ^242^E, ^262^P, ^274^K, ^286^VSD^289^R. The modified sequence of CoxD is being referred to as CoxD*. The 3D structure of CoxD was modeled with the automated web surfer ESyPred3D employing the CoxD* sequence shown and the 3D structure of BchI (1G8P) [31] from *Rhodobacter capsulatus* as template. (A) Structure of CoxD showing the N-terminal Rossmann-fold domain (orange) with central β-sheet core (green) and the C-terminal α-helical domain (blue). (B) Positions of AAA+ domain key elements on CoxD: Walker A (red), Walker B (blue), sensor 1 (magenta), arginine finger (orange), sensor 2 (green), VWA-binding motif (cyan). (C) Inserts of the PS-II-insert clade in CoxD; helix-2-insert (orange), PS-I-insert (blue), PS-II-insert (green). (D) A model of the BchI hexamer of the Mg-chelatase complex (pdb 2X31) [48] showing the inserts of the PS-II-insert clade. The program CHIMERA was employed to show the different views and color coding [52].

### Electron Microscopy and Image Processing

For TEM imaging, 3.5 µl of refolded CoxD (10–20 ng µl^−1^ in 100 mM Tris/acetate (pH 8.0) were applied to freshly glow-discharged holey carbon grids (300 mesh R2/4 Quantifoil® grids, Quantifoil Micro Tools GmbH, Jena, Germany) covered with an additional thin layer of continuous carbon. After 45 s at room temperature excess of liquid was blotted, and the sample was negatively stained using 2% (w/v) uranyl acetate for 15 s. Samples were evaluated at 28,500-fold magnification using a Philips CM100 electron microscope operated at 100 kV. For 2D analysis, data was collected using a Tecnai Spirit electron microscope equipped with a 2 k Eagle CCD™ camera (FEI, Eindhoven, The Netherlands) operated at 120 kV. Images were recorded automatically using the Leginon system [25] at a nominal magnification of 42,000-fold, resulting at a pixel size of 5.04 Å at the object scale, and a defocus range from −2 to −3 µm. Particles were selected semi-automatically using EMAN2 [26] after evaluation of the contrast transfer function using CTFFIND3 [27]. From projection images of the selected particles, 2D class averages were calculated using the SPIDER software package [28].

### Prediction of Secondary and Tertiary Structures

Secondary structures were predicted employing the online-server SABLE (Solvent AccessiBiLiEs of amino acid residues in proteins and improved prediction of secondary structures http://sable.cchmc.org/; [29]). Three dimensional structures were built employing the automated web surfer ESyPred3D (http://www.fundp.ac.be/sciences/biologie/urbm/bioinfo/esypred/) and the program package MODELLER [30]. The 3D structure of CoxD was modeled with the 3D structure of BchI (1G8P) from *Rhodobacter capsulatus* [31] as a template. Prior to modeling, the amino acid sequence of CoxD (295 aa) was adjusted to the sequence of BchI (350 aa) by taking the following steps resulting in the sequence referred to as CoxD* (Fig. 1A): Elongation of the N-terminal loop of CoxD through the addition of the sequence MTTAVARLP of BchI; insertion of the α1-β1 β-hairpin (32 aa) of BchI (Fig. 1B) after G^61^ of CoxD; deletion of 25 aa (which are absent from BchI) in the H2-insert of CoxD (Fig. 1B); integration of the sequence FLEEWRPKDMDIRN of BchI into the CoxD PS-II insert after I^226^. The predicted secondary structures of CoxD before and after adjustment showed same types and arrangement of secondary structural elements.

### Miscellaneous Methods

Bacteria were disintegrated by 5–15 passages through a French pressure cell (American Instruments, Silver Springs, MA, USA) at maximum pressure. Subsequent centrifugation (9,000×*g*, 30 min, and 4°C) yielded the cell free crude extract.

Samples for analysis on SDS (sodium dodecyl sulfate)-PAGE (polyacrylamide gel electrophoresis) were prepared by boiling for 4 min in 160 mM Tris/HCl (pH 6.8) containing 1% (w/v) SDS and 2.5% (w/v) 2-mercaptoethanol. Protein samples separated by SDS-PAGE [32] were either stained for protein with Coomassie Brilliant Blue R-250 or electro-blotted onto a polyvinylidene fluoride membrane (PVDF, Roth, Karlsruhe, Germany). Blots were subjected to N-terminal sequencing or immunodetection with rabbit IgG antibodies directed against CoxD [2]. Quantitation of Coomassie stained protein in PAGE gels was performed densitometrically employing the program ImageJ [33]. Protein was estimated employing the binding of Coomassie Brilliant Blue (Bradford [34]) or the Biuret-assay (Beisenherz [35]). Alternatively, CoxD was quantitated by its UV-absorption [ε_280_ (CoxD) = 30.035 mM^−1^ cm^−1^]. All chemicals employed were of analytical grade and purchased from usual commercial sources.

For sequence alignments the UVa FASTA Server (fasta.bioch.virginia.edu/fasta_www2/fasta_list2.shtml) was employed.

## Results

### Translation and Subcellular Localization of CoxD in *O. carboxidovorans*

The clustered genes *coxD*, *coxE* and *coxF*, which are essential for the posttranslational maturation of the [CuSMoO_2_] active site cluster of CO dehydrogenase [2], [9], are CO-specifically transcribed [8], [2]. Also the translation of CoxD required the presence of CO and was not apparent in bacteria grown with H_2_ plus CO_2_ or with organic substrates. CoxD was synthesized under chemolithoautotrophic conditions with CO as a sole source of energy and CO or CO_2_ as carbon source (Fig. 2, lane A) or with H_2_ as energy source and CO_2_ as carbon source in the presence of CO as an inducer of CoxD biosynthesis (Fig. 2, lane B). Under inducing conditions, CoxD was also synthesized in the mutants *coxE* and *coxF* (Fig. 2, lanes E and F), but not in the *coxD* mutant (Fig. 2, lane D). Although CoxD is devoid of transmembraneous segments (Fig. 1), it appeared insoluble (Fig. 2, lanes P) and was consistently absent from the cytoplasmic fraction (Fig. 2, lanes S). In addition, CoxD was firmly anchored to the cytoplasmic membrane of *O. carboxidovorans* as is evident from the requirement of anionic detergents (e.g. 2% w/v sodium dodecyl sulfate, 2% w/v N-lauroylsarcosine) for solubilization (data not shown). We have not been able to identify suitable detergents and conditions to solubilize CoxD in a folded state from membranes of its native host *O. carboxidovorans*. Tween 20, Triton X-100, *n*-dodecyl-β-D-maltoside, and *n*-octyl-β-D-glucopyranoside were not effective in the removal of the protein from cytoplasmic membranes. CoxD is only a minor component in the cytoplasmic membrane of *O. carboxidovorans* (<0.1% of the total membrane protein fraction). Facing this situation, attempts were made to overexpress CoxD in *E. coli*, which was anticipated to be more convenient and to give better yields of functional and stable protein than detergent solubilization of native CoxD from cytoplasmic membranes of *O. carboxidovorans*.

### Refolded Recombinant CoxD is Soluble and Includes Secondary Structural Elements

Large quantities of recombinant CoxD were produced in *E. coli* K38 pGP1-2/pETMW2. The protein entirely appeared in inclusion bodies, which might be ascribed to its high expression level. Adjustments of the bacterial cultivation to turn down the expression (e.g. low temperature, decreasing the IPTG-concentration) did not lead to a soluble product. We thus decided to take advantage of the high productivity of *E. coli* under the established conditions and of the high degree of purification of the recombinant CoxD in inclusion bodies. The molecular mass of the recombinant CoxD polypeptide of 33 kDa apparent from SDS-PAGE (Fig. 3, lanes 1, 3 and 5) matches the mass of 33.264 kDa deduced from the amino acid sequence [36], [7]. Densitometry of crude extracts subjected to SDS-PAGE revealed that CoxD amounted to ∼18% of the total cell protein of *E. coli* (Fig. 3, lane 2). Pure CoxD (>95%) could be prepared from inclusion bodies (Fig. 3, lanes 3 and 4) employing the protocol detailed in Materials and Methods. The identity of CoxD was further substantiated by Western blotting with specific IgG antibodies which showed a precipitin signal corresponding to 33 kDa (Fig. 3, lane 5) and N-terminal sequencing (Fig. 3, lane 3; MRHHAERDKV), which matched the amino acid sequence deduced from the nucleotide sequence of *coxD* (Fig. 1).

Refolding of CoxD from inclusion bodies could be achieved by solubilization with urea followed by stepwise dilution using the protocol detailed in the methods section. The absence of any turbidity in the absorption spectra of CoxD recorded at the beginning and at the end of the refolding process indicated that the protein did not precipitate under these conditions (Fig. 4A). CoxD kept in refolding buffer (*cf.* methods section) at 4°C was perfectly stable for at least one month. Refolded CoxD kept in 20 mM Tris/HCl (pH 8.0) or in 100 mM Tris/acetate (pH 8.0) remained soluble and did not develop aggregates in the tested temperature range of 4–30°C. On the other hand, our research was hampered to a certain extent by a pronounced sensitivity of refolded CoxD to different salts and chemical compounds which led to precipitation of CoxD within minutes to hours (more than 5 to 10 mM of MgCl_2_, CaCl_2_, NaCl, ATP, or MgATP). CoxD kept at pH 8–10 in 20 mM Tris/HCl containing 10 mM MgCl_2_ at 25°C was completely precipitated after 20 min.; at 4°C the stability of CoxD could be significantly improved, and complete precipitation took about 10 hours. To maintain CoxD in solution it was also essential to add the protein to the salt solution and not *vice versa*.

The presence of secondary structural elements in CoxD solubilized from inclusion bodies and refolded was apparent from circular dichroism (CD) spectroscopy (Fig. 4). The CD of refolded CoxD in the absence of MgATP or MgATP-γ-S (first mention) or of CoxD treated with MgATP-γ-S (second mention) revealed α-helices (20/15%), β-sheets (29/33%), turns (12/11%), and unordered structures (38/40%). Obviously, there is a disagreement of secondary structure in CoxD determined by CD (Fig. 4) or suggested by secondary structural prediction shown in Fig. 1 (47% α-helices, 11% β-sheets, 42% unordered) which is not cured by MgATP-γ-S. A similar phenomenon has been observed before with the eukaryotic chaperone Hsc70 [37]. CD of Hsc70 revealed a content of 15% α-helix, whereas the secondary structural prediction suggested 30% α-helix. This conformation, containing less secondary structure, was assumed to present Hsc70 in a molten globule state. CoxD and Hsc70 share some characteristic properties: Both are ATP-dependent chaperones which act on proteins in a posttranslational fashion and interact with membranes. It is tempting to assume that the secondary structure alterations apparent from the CD of refolded CoxD (*i.e.* less α-helix and more β-sheet), present a molten globule state or an intrinsically disordered state in coexistence with CoxD refolded into the ordered structure. The inspiring review article by Dunker et al. [38] gives examples of proteins which suggest that native protein structure can correspond to all three states (ordered, molten globule, and random coil) and that protein function can arise from any of the three states and their transitions. Whether the CoxD functions (*i.e.* ATPase activity, oligomerization) can arise from any of these states remains to be elucidated.

### NTPase Activity of CoxD

Refolded CoxD displayed ATP-hydrolyzing activity, which was apparent in Tris/acetate but not in Tris/HCl. ATPase activity strictly required the presence of Mg^2+^-ions (Fig. 5A), however, due to the instability of refolded CoxD at the assay temperature of 25°C the concentration should be below 10 mM. Excess EDTA (∼ 10 mM) was inhibitory to the reaction, presumably because of its ability to remove Mg^2+^ through complex formation. Concentrations of Mg^2+^-acetate or Na_2_ATP exceeding 2 mM led to precipitation of CoxD. For these reasons, NTPase activity assays were routinely supplied with 1 mM of each compound. Under these conditions, CoxD revealed NTP-hydrolyzing activities of 9.14±0.53 nmol P_i_ min^−1^ mg of protein^−1^ formed from ATP (Fig. 5C) and 0.2 nmol P_i_ min^−1^ mg of protein^−1^ formed from GTP (Fig. 5E). ATP was cleaved into equimolar amounts of ADP and P_i_ (Fig. 5B). ATP-hydrolysis by CoxD followed Michaelis-Menten kinetics (Fig. 5D). Depending on the method of calculation (non linear regression, Hanes and Woolf, Lineweaver-Burk) the K_M_ values (mM ATP) and V_max_ values (nmol ADP min^−1^ mg^−1^) were 0.58 and 17.72, 0.63 and 18.18, and 0.85 and 21.9, respectively. From this data a mean K_M_ of 0.69±0.14 mM ATP and a mean V_max_ of 19.3±2.3 nmol ATP hydrolyzed min^−1^ mg^−1^ can be calculated. ATP-γ-S is a slowly hydrolysable analogue of ATP. It very effectively competed with ATP for CoxD (Fig. 5F). In contrast, GTP apparently did not compete significantly with ATP at the active site of CoxD.

### Oligomerization of CoxD

AAA proteins function as oligomers, in most instances by forming hexameric rings [39]. BchI of the *R. capsulatus* Mg-chelatase produced in *E. coli* revealed in electron microscopic single particle analysis circular complexes with six or less protomers per particle which led to a hexameric model of BchI at atomic resolution [40]. Therefore, it was tempting to assume that CoxD would also form oligomeric complexes. Sucrose density gradient centrifugation showed a broad distribution of CoxD in the mass range of 38 to 343 kDa (Fig. 6A). The shoulders (referred to as a and b in Fig. 6A) and a prominent peak (c in Fig. 6A) correspond to masses of 59 kDa (a), 123 kDa (b), and 211 kDa (c). On the basis of the mass of CoxD (33.267 kDa) deduced from its amino acid sequence, the structural organization of a, b, and c can be interpreted as dimeric, tetrameric and hexameric, respectively. All experiments in Fig. 6 show that the 6-mer represents the most prominent oligomerization state of freshly refolded CoxD. Interestingly, only the dimers and the hexamers could hydrolyze ATP (Fig. 6A). On a short term basis, the CoxD hexamer remained stable and did not dissociate (Fig. 6C). However, diluted solutions of the 6-mer nearly completely decomposed into the 2-mer (2×33 kDa) after several weeks of incubation in the refrigerator (Fig. 6B). The process was significantly slower in CoxD solutions with high protein content (Fig. 6B). The reaction could be reversed by MgATP (Fig. 6D) or MgATP-γ-S (Fig. 6E) within few hours. The 6-mer (main peak) and a small amount of protein outside the separation range of Sephacryl S-300 could also be demonstrated by gel filtration of freshly refolded CoxD (Fig. 6F).

This data prompted us to study the structural organization of CoxD using transmission electron microscopy in combination with the single particle approach. TEM images of negatively stained preparations of CoxD indeed revealed distinct particles within a size range of 10–16 nm (Fig. 7A). This is in good agreement with the size of other protein complexes with a molecular mass of approximately 200 kDa such as for example the cGMP phospodiesterase 6 from the visual transduction cascade [41]. However, some smaller fragments as well as particles exhibiting a more elongated shape were also present in the raw images. After semi-automated particle picking, 19,091 raw particles were used for a reference-free alignment and calculation of class averages using the k-means clustering algorithm implemented in the SPIDER software package. Stable class averages were obtained using 30 classes (Fig. 7B). Reducing the number of classes did not prevent the occurrence of nearly identical views. However, visual inspection as well as sub-classification showed that classes representing more elongated features (classes 4, 8, 10, 12, 14 and 15) were clearly heterogeneous (data not shown). Particles from these classes were rejected and new class averages were calculated using the remaining 15,876 particles. Stable class averages were obtained now using only 20 classes (Fig. 7C). Classification with more classes reproduced only identical views whereas characteristic views disappeared when using fewer classes.

Visual inspection of the classes showed, that particles within individual classes were reasonably homogeneous (Fig. 7D). Sub-classification of particles from individual classes did not educe significantly different new views. The obtained class averages demonstrate reproducible substructure. They clearly establish an oligomeric organization of CoxD. Due to the size of the particles, some classes can be interpreted in terms of a hexameric organization. Although samples used for TEM analysis have been taken from the peak fraction after sucrose density gradient centrifugation which mainly contains hexamers of CoxD (c in Fig. 6A), TEM images show that the heterogeneity of the particles is still too high to determine the organizational state unambiguously (Fig. 7A). Therefore, it is impossible to distinguish between the views of complexes of different sizes (e.g. dimers, tetramers, and hexamers) and/or different orientations. Additional experiments are required to isolate complexes of unique sizes as a prerequisite for further 3D structural analysis using single particle techniques.

## Discussion

### Direct Evidence Identifies CoxD as an ATPase

CoxD has been predicted as an AAA+ ATPase chaperone related to BchI of Mg-chelatase [2]. Mutational studies have established that it functions in cooperation with CoxE and CoxF in the biosynthesis of the [CuSMoO_2_] cluster in the active site of CO dehydrogenase through the stepwise introduction of S and Cu in the apo-enzymés [MoO_3_] center [2], [42], [9]. Particularly the Walker A and Walker B motifs on the amino acid sequence of CoxD suggest a P-loop ATPase with the ability to bind and hydrolyze ATP (Fig. 1). For AAA+ proteins, nucleotide hydrolysis is the major event that promotes their specific biological activities [49]. Employing refolded recombinant CoxD produced in *E. coli*, we provide direct evidence which identifies CoxD as a true ATPase. CoxD hydrolyzed MgATP, yielding MgADP and inorganic phosphate at a 1∶1∶1 molar ratio. The activity was strictly dependent on Mg^2+^-ion. The highest ATPase activities of CoxD preparations (ranging from 19 to 44 nmol P_i_ min^−1^ mg^−1^) favorably compare to the activities of recombinant ChlI from *Arabidopsis thaliana* (55 nmol min^−1^ mg^−1^, [43]), recombinant BchI from *Rhodobacter sphaeroides* (108.3 nmol P_i_ min^−1^ mg^−1^ [44]) or recombinant ChlI from *Synechocystis* PCC6803 (147.5 nmol P_i_ min^−1^ mg^−1^ [45]). That CoxD is an ATPase is further corroborated by the action of ATP-γ-S, a slowly hydrolyzed analog of ATP, which inhibited the hydrolysis of MgATP by CoxD (Fig. 5F). CoxD showed only slight MgGTPase side activity of less than 2% of its MgATPase activity (Fig. 5E).

CoxD appears exclusively in the cytoplasmic membrane of *O. carboxidovorans* cultivated in the presence of CO (Fig. 2) and resembles in this aspect many ATPases, including the human Cu-transporting P-ATPase ATP7A [46]. The very low overall sequence similarity of CoxD and Cu-transporting P-ATPases [3.4% with human ATP7A and 3.1% with human ATP7B [46]] along with the absence of membrane-spanning domains make a function of CoxD under its own steam in the transport of Cu^+/++^-ion into the bacterial cell unlikely. In addition, the genome of *O. carboxidovorans* [47] reveals three Cu-translocating P-type ATPases on the chromosome (open reading frames: OCAR_7144, OCAR_7520, and OCAR_7762) which potentially could account for Cu-transport.

### The 6-mer and the 2-mer Represent the Most Characteristic Oligomeric States of CoxD

According to Vale [39] AAA-proteins function as oligomers, in most cases by forming hexameric rings. The BchI subunits of Mg-chelatase are arranged into a trimer of dimers within a ring structure [48], [15]. Although BchI has been described as a 240-kDa hexamer, complexes with less than six protomers per particle have also been observed which has been ascribed to the fragility and dynamic nature of the BchI-complexes [40]. Normally, ATP binding and hydrolysis in AAA+ family members takes place at the interfaces between subunits that form the oligomeric ring structures [39]. ATP has also been implicated as an important factor in stabilizing these structures [48]. Freshly refolded CoxD forms mainly 6-mers which dissociate into 2-mers in a reaction which can be reversed by ATP (Fig. 6). Apparently, ATP serves two functions as it is a substrate of CoxD and also supports the oligomerization of 2-mers to 6-mers. The transcriptional activator PspF_1–275_ exists in equilibrium between dimeric and hexameric states, where hexamerization is favored at higher PspF_1–275_ concentrations or by the presence of ATP or ADP [50]. Sucrose density gradient centrifugation and gel filtration (Fig. 6) along with single particle electron microscopy (Fig. 7) clearly indicates that CoxD forms complexes also in the absence of nucleotides. The complexes represent various multimeric states, namely dimers, tetramers and hexamers. Of these, only the dimers and the hexamers act as ATPases (Fig. 6A).

### A Model of the CoxD Three-dimensional Structure

In essence CoxD is a complete AAA+ domain composed of an N-terminal Rossmann-fold subdomain with central β-sheet core and a C-terminal α-helical subdomain (Fig. 8A). It has the key elements of an AAA+ domain (Fig. 8B) in the same arrangement and at same positions as in BchI [31], [48] and displays the characteristic inserts of the PS-II-insert clade [13] (Fig. 8C). The model of the BchI hexamer of the Mg-chelatase complex shows a trimer of BchI dimers (Fig. 8D). Application of this model to CoxD would explain why the protein consistently appeared dimeric, tetrameric or hexameric, but never monomeric or trimeric (Fig. 6). It is also interesting that the helix-2-insert and the PS-I-β-hairpin are exposed towards the inner side of the central pore of the hexamer, whereas the PS-II-helix surrounds the hexamer at the outside (Fig. 8D). However, although CoxD definitely forms 6-mers, it requires further substantiation whether or not these arrange as ring-like structures as with other AAA-proteins [39]. In conclusion, CoxD is a membrane-bound Mg^2+^-dependent ATPase, which can undergo hexameric-dimeric interconversion and is a new addition to the AAA+ protein family.

### Possible Function of CoxD in the Assembly of the [CuSMoO_2_] Active Site Cluster of CO Dehydrogenase

Up to date two specific functions can be ascribed to CoxD. One is the introduction of sulfur into the active site of apo-CO dehydrogenase, and the other is that of an ATP-dependent chaperone. Insertional mutagenesis of *O. carboxidovorans* has established that the CoxD protein cooperates with CoxE and CoxF in the post-translational biosynthesis of the [CuSMoO_2_] active site cluster of CO dehydrogenase [2]. CoxD is associated with the cytoplasmic membrane ([2] Fig. 2). This implies that the maturation of CO dehydrogenase proceeds at the membrane. The specific, CO-dependent transcription of the *coxD* gene along with the CO dehydrogenase structural genes *coxMSL* is in accordance with its role in the assembly of the enzymes bimetallic active site. CoxD is engaged in the stepwise introduction of S and Cu in the apo-enzymés [MoO_3_] site [2]. The apo-CO dehydrogenase of the *coxE* mutant of *O. carboxidovorans* was complete in cyanolysable S, but Cu was absent [9]. Obviously, CoxD is involved in the transfer of the sulfane sulfur to the [MoO_3_] site, a process which is independent of CoxE and CoxF. Whether CoxD itself is the sulfur transferase, or whether functions of CoxD are required for the action of a separate sulfur transferase, must await further studies. The subsequent introduction of Cu into the [MoO_2_S] site requires the additional functions of CoxE and CoxF ([9], A. Pelzmann and F. Mickoleit, personal communication). In other words, the concerted actions of CoxD, CoxE, and CoxF might be referred to as a Cu-chelatase. The fact that CoxD on its own cannot transfer Cu agrees with the absence of Cu-binding motifs of Cu-chaperones [51] on the CoxD sequence (Fig. 1).

This report has established CoxD as a AAA+ ATPase (Figs. 5 and 6A) which corroborates its function as a chaperone suggested by bioinformatic data ([2], Figs. 1 and 8). Although there is no evidence available at present, two targets of CoxD chaperone action can be imagined. One is apo-CO dehydrogenase in which the active site is accessible from the outside through a narrow channel that is 17 Å deep with an average diameter of 7 Å [3]. Through the introduction of partial conformational changes into apo-CO dehydrogenase, CoxD might render the active site accessible for the biosynthetic machinery required for maturation of the bimetallic cluster. Alternatively, the chaperone action of CoxD could be directed to hypothetical transferases of S (or Cu) in a threading mechanism [49]. Such a mechanism would expose a short polypeptide chain carrying an active cysteine persulfide which is thread through the pore of the CoxD hexamer and then presented to the active site of apo-CO dehydrogenase. It can be imaged that such a polypeptide chain can enter the active site through the substrate channel. The accessibility of the CO dehydrogenase active site for outside molecules is much better than deduced from the crystal structure [3]; examples are n-butylisocyanide [4], coenzyme A, and glutathione (O. Kreß, personal communication). In addition, the *in vitro* reconstitution of the CO dehydrogenase active site through the supply of sulfide first and subsequently of Cu(I) from [Cu(I)(thiourea)_3_]Cl, under reducing conditions [42] readily proceeds without the requirement of a chaperone or any measure to unfold the protein.
